# Characterization and phylogenetic relationship of the complete mitochondrial genome of Black-cheeked Lovebird, *Agapornis Nigrigenis*

**DOI:** 10.1080/23802359.2019.1677195

**Published:** 2019-10-15

**Authors:** Yun-Xia Chen, Ya-Lin Huang, Jia-Qi Liu, Yong-Wu Zhou, Sen-Lin Hou

**Affiliations:** a Nanjing Forest Police College, Nanjing, China;; b Key Laboratory of Wildlife Evidence Technology State Forest and Grassland Administration, Nanjing, China;; c Beijing Forestry University, Beijing, China

**Keywords:** Black-cheeked Lovebird, *Agapornis nigrigenis*, mitochondrial genome

## Abstract

The complete mitochondrial genome of *Agapornis nigrigenis* was obtained using Sanger sequencing. The mitochondrial genome from *A. nigrigenis* is 16,718 bp in length. Its genome with an overall GC content of 48.86%, contained 22 transfer RNA genes, 13 protein-coding genes, 2 ribosomal RNA genes, and 1 control region. The phylogenetic position of *A. nigrigenis* based on the 18 parrots species showed it was sister to the same genus specie *Agapornis roseicollis*. The results obtained here can contribute to molecular evolution and phylogeny of parrots further.

Black-cheeked Lovebird (*Agapornis nigrigenis*) belongs to the family Psittacidae (Aves, Psittaciformes), known as its affectionate nature. This species is mainly distributed in southwestern Zambia, inhabits shrubs of Acacia, thorny plains, and the open grass between 600 and 1000 m (Soobramoney and Perrin 2014; IUCN 2016). Because of the bright and colourful feathers, this specie is now being bred in captivity for the pet trade and is one of the most popular parrots in the Chinese pet market (Warburton and Perrin 2006; Frynta et al. 2010).

Captive-bred specimen of *A. nigrigenis* was sampled from the Nanjing Hongshan Forest Zoo (N32°09′, E118°80′), Jiangsu province, China. Whole blood sample for genomic DNA extraction was collected from the individual and stored in the Forest Police Forensic Centre of State Forestry Administration (Accession S2019J1101202). DNAiso reagent (Takara) was used for genomic DNA extraction, a set of primers were designed for polymerase chain reaction amplification, and Sanger sequencing was performed to obtain genome information.

The complete mitochondrial genome (GenBank accession: MN481405) of *A. nigrigenis* is 16,718bp in length. The overall base composition of the genome is 21.52% T, 34.57% C, 29.62% A, 14.28% G, exhibiting an A + T bias (51.14%). The structure and gene arrangement of the mitogenome is highly conserved and identical to that of most avian species, consisting 13 protein-coding genes, 22 transfer RNA genes, 2 ribosomal RNA genes, and 1 control region. (Eberhard and Wright 2016; Liu, Cheng-He, et al. 2019; Liu, Jin, et al. 2019).

To confirm the phylogenetic position of *A. nigrigenis*, 18 complete mitochondrial genome sequences of Psittacidae were aligned using ClustalX and neighbour-joining (NJ) analysis was conducted using MEGA 7.0, with 1000 bootstrap replicates (Kumar et al. 2016). The phylogenetic NJ tree showed that *A. nigrigenis* was sister to the same genus specie *Agapornis roseicollis* (Figure 1). The mitogenome information obtained here will be useful for the conservation and phylogeny of *A. nigrigenis*.

**Figure 1.**
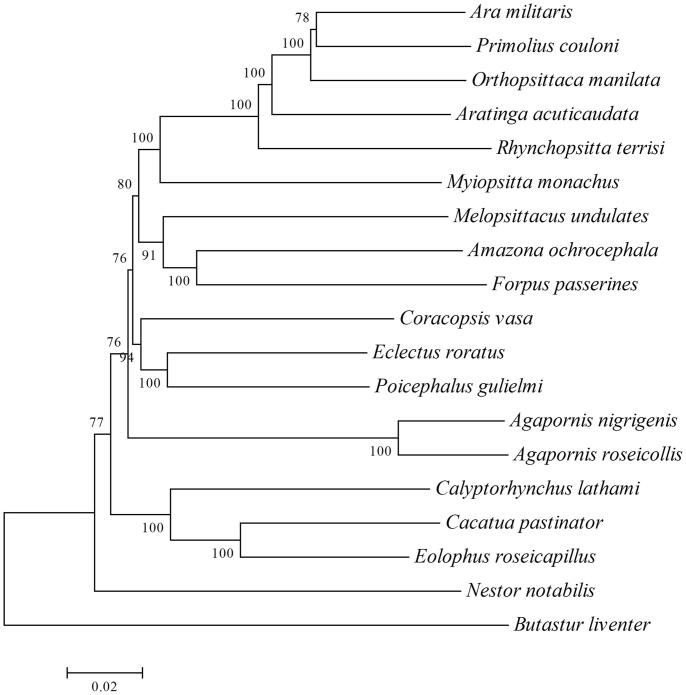
Neighbour-joining phylogenetic tree based on the complete mitogenomes of 18 Psittacidae species, constructed using MEGA 7.0. Eighteen parrots species mitochondrial genomes have been deposited in the GenBank, the accession numbers are as follows: *Agapornis roseicollis* EU410486.1, *Amazona ochrocephala* KM611467.1, *Ara militaris* KM611466.1, *Aratinga acuticaudata* JQ782214.1, *Butastur liventer* AB830617.1, *Cacatua pastinator* NC_040142.1, *Calyptorhynchus lathami* JF414241.1, *Coracopsis vasa* KM611468.1, *Eclectus roratus* KM611469.1, *Eolophus roseicapillus* NC_040154.1, *Forpus passerines* KM611470.1, *Melopsittacus undulates* EF450826.1, *Myiopsitta monachus* NC_027844.1, *Nestor notabilis* MH133967.1, *Orthopsittaca manilata* KJ579139.1, *Poicephalus gulielmi* MF977813.1, *Primolius couloni* KF836419.1, *Rhynchopsitta terrisi* KF010318.1.
